# Development and performance of prototype serologic and molecular tests for hepatitis delta infection

**DOI:** 10.1038/s41598-018-20455-5

**Published:** 2018-02-01

**Authors:** Kelly E. Coller, Emily K. Butler, Ka-Cheung Luk, Mary A. Rodgers, Michael Cassidy, Jeffrey Gersch, Anne L. McNamara, Mary C. Kuhns, George J. Dawson, Lazare Kaptue, Birgit Bremer, Heiner Wedemeyer, Gavin A. Cloherty

**Affiliations:** 1 Abbott Laboratories, Abbott Park, IL USA; 2 Université des Montagnes, Bangangté, Cameroon; 3 Department of Gastroenterology, Hepatology and Endocrinology, Hannover Medical School, Hannover, Germany

## Abstract

Worldwide, an estimated 5% of hepatitis B virus (HBV) infected people are coinfected with hepatitis delta virus (HDV). HDV infection leads to increased mortality over HBV mono-infection, yet HDV diagnostics are not widely available. Prototype molecular (RNA) and serologic (IgG) assays were developed for high-throughput testing on the Abbott *m*2000 and ARCHITECT systems, respectively. RNA detection was achieved through amplification of a ribozyme region target, with a limit of detection of 5 IU/ml. The prototype serology assay (IgG) was developed using peptides derived from HDV large antigen (HDAg), and linear epitopes were further identified by peptide scan. Specificity of an HBV negative population was 100% for both assays. A panel of 145 HBsAg positive samples from Cameroon with unknown HDV status was tested using both assays: 16 (11.0%) had detectable HDV RNA, and 23 (15.7%) were sero-positive including the 16 HDV RNA positive samples. Additionally, an archival serial bleed panel from an HDV superinfected chimpanzee was tested with both prototypes; data was consistent with historic testing data using a commercial total anti-Delta test. Overall, the two prototype assays provide sensitive and specific methods for HDV detection using high throughput automated platforms, allowing opportunity for improved diagnosis of HDV infected patients.

## Introduction

Hepatitis delta virus (HDV) is classified as a defective virus as viral particle assembly requires packaging of RNA into the HDV encoded capsid (HDAg) which is associated with an envelope protein provided by the hepatitis B virus (HBV) surface antigen (HBsAg, L, M, and S) proteins^1–3^. Infection is established simultaneously with HBV or through superinfection of an HBV positive individual. HDV infection is associated with elevated risk of chronic hepatitis, accelerated liver disease, and poorer prognosis than HBV infection alone^4,5^. Despite global HBV vaccination programs, HDV infection is on the rise in different geographies^3,6–8^. The estimated global burden of HDV is 15–20 million (5% of HBV infections) with an increased mortality rate among dually infected individuals^9^.

HDV exhibits a high degree of genetic heterogeneity, with intra-genotypic divergence less than 16% and approximately 19–38% between genotypes^6,10–12^. To date, there are eight proposed genotypes for HDV, with genotype 1 the most globally prominent^12,13^. The genotype 3 clade of HDV, endemic to South America, exhibits the greatest divergence with approximately 70% similarity to the other genotypes^12,14^. It has been proposed that genotype-dependent variations in disease progression exist^15^, but needs to be further evaluated. The presence of quasispecies is noted within individual patients and is thought to result from poor editing ability of the RNA polymerase involved in HDV replication^11,16,17^. The most conserved region of HDV genome is within the ribozyme^10^, which has served as a target for molecular based assays^18–21^.

Because of the higher risk of developing liver-related complications resulting from HDV superinfection, HDV diagnostic testing is recommended for all patients with chronic HBV infection^6,8^. Serologic testing is suitable for initial evaluation with reflex RNA testing to differentiate active from resolved infections. The current diagnostic landscape includes a need for highly sensitive and specific serologic and molecular assays for HDV detection: however, in resource limited areas availability is scarce and there is no standardized molecular assay for HDV detection globally available^8,22^. Further, many RNA tests work well for genotype 1, but not for other genotypes^23^. In 2013, the World Health Organization (WHO) established an international standard for HDV RNA, a genotype 1 strain, that can be used in nucleic acid amplification technique (NAT)-based assays^22^, but assessment of assay performance using the WHO standard may not capture the challenge of detecting diverse HDV strains.

Herein, we describe the development of prototype serologic (anti-HDV IgG) and molecular (quantitative reverse-transcription polymerase chain reaction, qRT-PCR) assays to detect HDV infection, adapted for high-throughput screening on the Abbott ARCHITECT (serology) and *m*2000 (molecular) platforms, respectively. The molecular assay targeted the conserved ribozyme region and was challenged by testing *in vitro* transcripts representative of the diversity within published HDV sequences. A panel of HBV carriers from Cameroon (n = 145) was tested and assay performance was validated by testing with two additional published assays^20,24^. A serology assay for the detection of HDV IgG was developed using peptides from the large antigen, and regions having linear epitopes were identified by testing a peptide scan library. Testing with both HDV molecular and serologic prototypes revealed all HDV RNA positives detected were antibody positive supporting serologic testing for routine diagnosis of HDV.

## Results

### Development of HDV molecular assay

**Figure 1:**
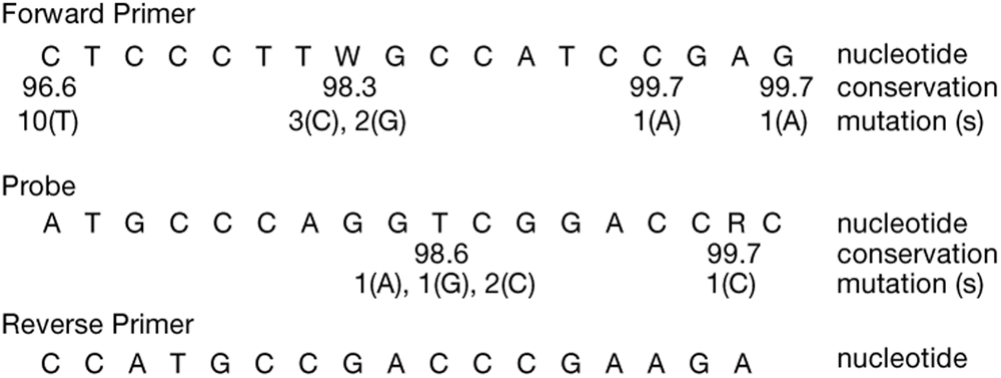
Diversity of primers and probe binding region. Complete HDV genome sequences (n = 291) were aligned using MUSCLE. Shown are primers (W = A, T) and probe (R = A, G) sequences. The percent conservation is indicated for nucleotide positions with mutation, along with the corresponding number of sequences with the indicated mutation. Where no number is listed, the conservation is 100% at the nucleotide position.

A qRT-PCR assay for detection of HDV RNA was developed using sample extraction and amplification protocols on the automated Abbott *m*2000 platform. Primers and probe targeted the conserved ribozyme region. HDV genome sequences from genotypes 1–8 (n = 291) were aligned and the targeted regions were analyzed for genetic diversity (Supplemental Table S1). Oligonucleotide sequences were 100% identical to targets in 269 of 291 (92.4%) HDV genotype 1–8 sequences (Fig. 1). The 22 sequences that harbored point mutations (Supplemental Table S1) included divergent isolates classified as genotype 1, 2, 5, 7, 8 and unclassifiable. The majority of sequences (20/22) had a single mutation while 2 sequences (LT604971.1, GU177114.1) had two mismatches in either the forward primer or probe regions. The most common mutation was a cytosine to thymine substitution at the 5′ end of the forward primer binding site found in 10 of 22 sequences (Fig. 1).

**Figure 2:**
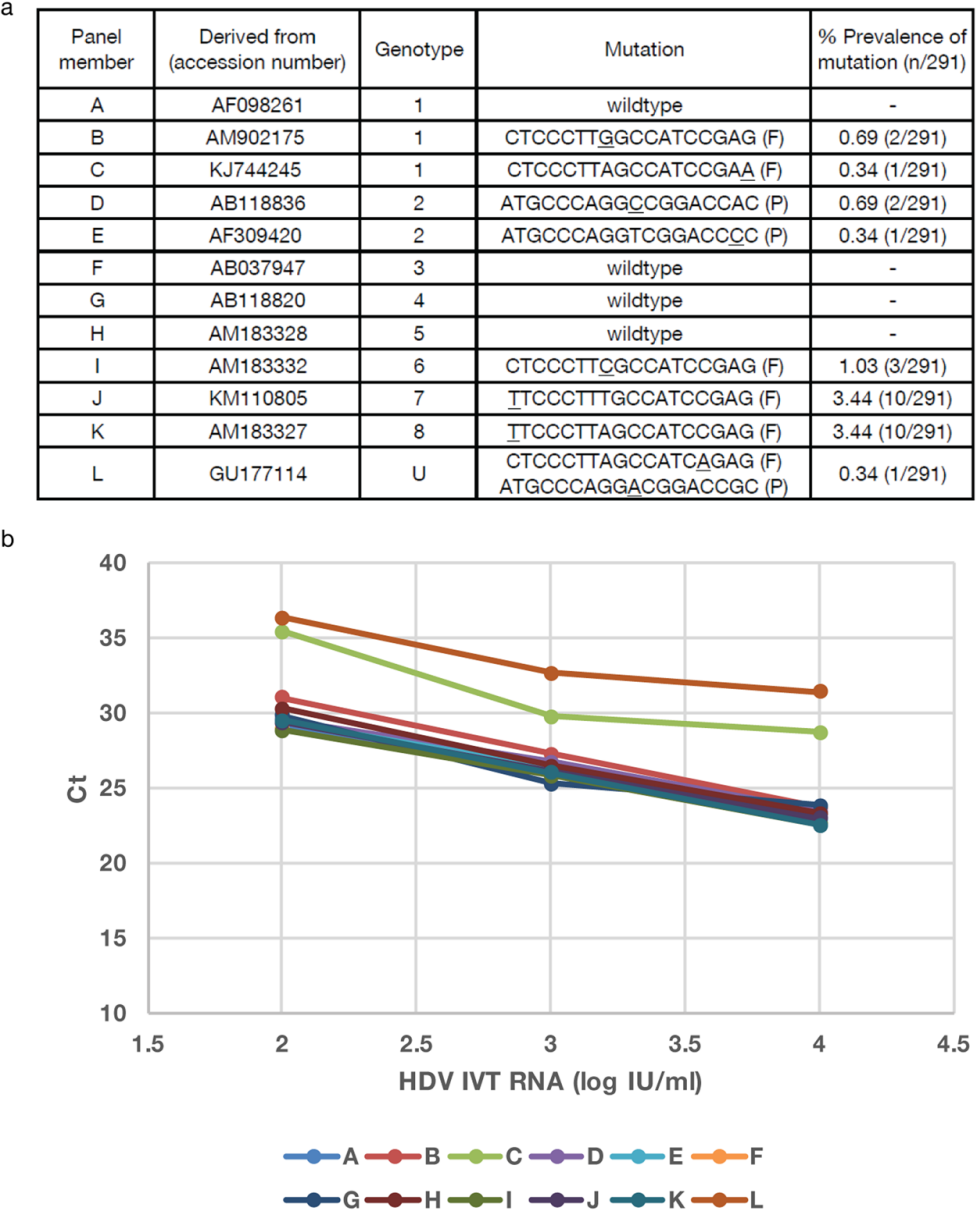
Evaluation of mutation challenge panel in molecular assay. A. *In vitro* transcribed RNA mutation panel. Indicated are the Genbank accession numbers, genotype, and mutations in each *in vitro* transcribed RNA. Frequency of mutation in the 291 sequences analyzed is represented by percent prevalence of mutation. U: unclassified, F: forward primer, P: probe. B. *In vitro* transcribed RNAs were diluted from 10^4^ to 10^2^ IU/ml and tested in the HDV molecular assay. Samples were tested in triplicate for each dilution and the average Ct value was plotted for each panel member.

A panel of 12 *in vitro* transcribed RNAs representing identified point mutations and the different HDV genotypes was designed to challenge the molecular assay (Fig. 2A). Targets were tested at 10^4^, 10^3^, and 10^2^ copies/mL, and all *in vitro* transcribed RNAs in the challenge panel were detected. The most common mutation (C to T in the forward primer) was present in 2 *in vitro* transcribed RNAs and did not impact detection (Fig. 2B). The most divergent panel member L, representing GU177114.1 an African isolate of unclassified genotype, had mismatches in both the forward primer and probe was detectable albeit delayed.

### Evaluation of molecular assay performance

In order to determine assay sensitivity, dilutions of the WHO HDV standard (genotype 1) were evaluated, and the provisional limit of detection (LOD) was determined to be 5 IU/ml (Table 1). Reproducibility, linearity and amplification efficiency of the assay was evaluated on 10-fold serial dilutions of the WHO HDV standard (5–50,000 IU/ml) (Table 2). Triplicate sets of dilution series (2 intra-run and 1 inter-run sets) showed low standard deviation (SD) in Ct values, demonstrating good inter- and intra-assay reproducibility (Table 2). The mean correlation coefficient (R^2^) of 0.9983 demonstrated linearity across the tested range; the mean slope of −3.28 showed high amplification efficiency (Table 2).

**Table 1:** Limit of detection and reproducibility on low copy dilutions of the WHO HDV standard.

IU/ml	#tested	#detected	%detection	Median Ct	Intra-assay SD (mean)	Inter-assay SD (mean)
1.25	99	93	94	36.60	NA	NA
2.5	114	106	93	36.13	NA	NA
5	111	110	99	34.74	1.110	1.137
10	50	50	100	33.23	2.200	0.859
20	12	12	100	31.85	1.920	0.562

Sensitivity was determined by diluting the WHO HDV standard diluted in in HBV/HDV negative human plasma to the indicated IU/ml. Dilutions were tested at the indicated number of replicates; percent detection was determined based on the number of replicates detected. Reproducibility was evaluated for concentrations above the determined LOD; shown are the median Ct, average intra-assay standard deviation (SD) of Ct values, and inter-assay SD of Ct values.

**Table 2:** Linearity of HDV molecular assay on WHO HDV dilutions.

	Ct values					R^2^	slope
	5 IU/ml	50 IU/ml	500 IU/ml	5000 IU/ml	50000 IU/ml		
**Intra-run**							
Exp 1.	34.75	31.13	28.20	24.80	21.36	0.9992	−3.30
Exp 2.	34.98	31.01	28.10	25.10	21.66	0.9972	−3.25
**Inter-run**							
Exp 3.	34.23	30.97	28.09	24.60	21.01	0.9984	−3.28
Mean of Exp 1–3	34.65	31.04	28.13	24.83	21.34	0.9983	−3.28
SD of Exp 1–3	0.38	0.08	0.06	0.25	0.33	0.0010	0.03

Shown are three experiments of 10-fold dilution series of WHO HDV plasma standard (5–50000 IU/ml) tested in the Abbott molecular prototype assay. Correlation coefficient (R^2^) showing the linearity of each standard curve and slope denoting the amplification efficiency are given for each set of the serial dilutions.

Specificity of the molecular assay was determined by testing a panel of plasma donors sourced from the United States which were pre-screened for HBV (HBsAg, anti-HBc, and DNA), HCV (antibodies and NAT), HIV (antibodies and NAT) (n = 68). No false positives were observed: specificity was 100% (95% CI, 0.94 to 1.0) in this population.

### Identification of active HDV infection in HBsAg positive samples from Cameroon

The utility of the molecular assay to detect active HDV infection was evaluated by testing 145 HBsAg positive samples from Cameroon with unknown HDV RNA or antibody status. A total of 16 of 145 (11.0%) samples had detectable HDV RNA and viral loads ranged between 1.49 to 7.90 log IU/ml using the prototype molecular assay (Table 3). In order to evaluate the range of genotypes detected in this small sample set, HDV RNA positive samples with viral load >4 log IU/ml with available volume were sequenced for phylogenetic analysis. Genotypes 1 (samples 1134, 515–74, 220–01, 1038–64, and CHU898) and 6 (sample 886–24) were identified among those sequenced.

**Table 3:** HDV RNA positive samples from Cameroon.

Sample	HDV VL (log IU/ml)	HBsAg S/CO^1^	HBV VL^2^ (log IU/ml)
220–01	4.86	4912.04	1.58
223–04	5.45	4368.49	1.63
1038–64	6.30	3594.27	<1.00
957	5.10	4923.90	1.55
1189	1.60	4126.70	<1.00
1190	1.49	4491.80	2.97
CHU898	3.97	4706.20	1.68
23–23	4.89	4808.00	1.66
515–74	3.18	4576.00	2.10
819–30	3.14	3065.60	2.82
CHU2831	2.77	5405.00	3.71
CHU2810	1.94	6231.67	<1.00
UNI22	7.90	2573.77	<1.00
886–24	5.76	5296.00	2.37
1134	7.20	4972.40	2.24
891–30	1.84	5016.00	6.37

^1^S/CO ≥1.00 is considered positive. Determined using the ARCHITECT HBsAg (1L80) or ARCHITECT HBsAg Qualitative (4P53) assays (Abbott Diagnostics, Abbott Park, IL).

^2^HBV viral load determined using the RealTi*m*e HBV viral load assay (Abbott Molecular Diagnostics, Des Plaines, IL).

### Confirmation of HDV RNA positives using published assays

In order to verify results, the 14 HDV RNA positive Cameroon samples identified above were subsequently tested with 2 other published HDV RNA assays, Assay 1^24^ and Assay 2^20^ (Supplemental Table S2), that were calibrated using the WHO HDV standard. Both comparator assays corroborated the HDV molecular assay prototype data on 10 of 14 samples (Table 4). Two samples (1189 and 1190) were RNA positive by the prototype assay but negative by both comparator assays (Table 4); these samples had relatively low viral loads using the prototype molecular assay (1.60 and 1.49 log IU/ml). Additionally, HDV RNA was detected in two samples (891–30 and 515–74) by the prototype assay and Assay 2, but were not detected by Assay 1 (Table 4). Overall, correlation of results between Assay 1 and the prototype assay was high (R^2^ = 0.79) in contrast to Assay 2 which showed low correlation with the other two assays (Supplemental Figure S1).

**Table 4:** Confirmation of HDV RNA positive samples using comparator assays.

sample ID	HBsAg S/CO	Abbott		Assay 1^24^		Assay 2^20^	
		Ct	Log IU/ml	Ct	Log IU/ml	Ct	Log IU/ml
819–30	3065.60	25.31	3.14	28.97	2.85	29.3	5.18
220–01	4912.04	19.7	4.86	29.74	2.65	31.28	4.62
223–04	4368.49	17.78	5.45	29.15	2.81	27.15	5.78
1038–64	3594.27	15.03	6.30	15.14	6.39	23.73	6.75
CHU898	4706.20	22.61	3.97	22.17	4.59	30.85	4.74
957	4923.90	18.91	5.10	25.23	3.81	34.85	3.68
1189	4126.70	30.31	1.60	neg	ND	neg	ND
1190	4491.80	30.68	1.49	neg	ND	neg	ND
1134	4972.40	12.08	7.20	15.3	6.35	30.09	4.95
UNI22	2573.77	9.82	7.90	15.64	6.58	31.00	4.7
891–30	5016.00	29.55	1.84	neg	ND	30.79	4.76
515–74	4576.00	25.17	3.18	neg	ND	27.07	5.81
23–23	4808.00	19.62	4.89	17.84	5.7	27.32	5.74
886–24	5296.00	16.77	5.76	15.09	6.4	25.13	6.35

ND = not detected.

Samples were extracted using a common protocol as described in the methods; amplification conditions specific to each assay were maintained for comparison (Supplemental Table S2). Shown are viral loads determined by standard curve analysis using the WHO HDV NAT standard.

### Development of serologic assay prototype to detect HDV IgG

**Figure 3:**
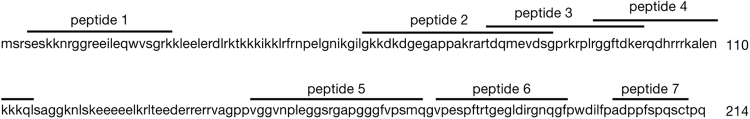
Peptides designed to HDV large antigen for serology assay. Consensus sequence of HDV large antigen from 230 sequences. Indicated are regions designed as peptides.

Alignment of HDV antigen sequences resulted in a consensus sequence which was used to design seven peptides spanning regions that were predicted to be antigenic, hydrophilic, and surface exposed (Fig. 3). An indirect IgG assay was developed using the HDV specific peptides on the Abbott Laboratories ARCHITECT platform to allow for high-throughput testing. HDV RNA positive samples identified with the prototype molecular assay were used to evaluate which peptides had value to detect HDV antibodies. All HDV RNA positive samples tested had detectable antibodies by at least 1 peptide, including the two HDV RNA samples with low viral load that were not detected in Assay 1 or 2 (Table 5). No single peptide detected all 16 HDV RNA positive samples; but peptides 2 and 4 (peptides derived from non-overlapping regions, Fig. 3) detected 15 and 14 samples, respectively, with peptide 2 showing the strongest signal to noise (S/N) values (Table 5).

**Table 5:** HDAg peptide reactivity of RNA positive samples.

	peptide						
	1	2	3	4	5	6	7
sample	S/N	S/N	S/N	S/N	S/N	S/N	S/N
220–01	1.31	**418.98**	2.58	**116.23**	**176.18**	**125.40**	1.41
223–04	0.53	**231.23**	1.10	0.94	3.52	1.65	1.89
1038–64	0.74	**169.01**	0.83	**134.97**	0.95	0.82	1.07
957	0.59	**399.58**	0.77	**111.50**	2.02	1.67	1.04
1189	4.54	**291.13**	0.85	9.01	**354.75**	0.98	2.39
1190	0.34	**394.08**	0.29	**14.68**	**31.29**	0.49	0.38
chu898	0.79	**245.33**	0.94	**81.38**	**37.79**	3.32	0.49
23–23	1.21	**310.30**	1.58	**73.48**	**24.02**	1.86	2.05
515–74	**11.38**	9.04	2.84	**88.26**	**16.72**	3.22	3.26
819–30	1.33	**293.45**	**243.75**	**130.41**	**24.57**	**21.64**	0.56
chu2831	0.26	**358.73**	0.32	**46.88**	0.38	0.28	0.38
chu2810	0.34	**415.47**	**16.22**	**333.99**	**338.98**	**415.47**	0.51
uni22	1.32	**65.83**	0.92	**23.52**	0.86	4.79	1.29
886–24	1.43	**486.37**	1.50	**252.95**	1.58	1.81	1.89
1134	2.21	**334.30**	**107.09**	**315.01**	1.95	2.25	2.16
891–30	2.13	**317.81**	**312.65**	**191.42**	**40.73**	**22.32**	0.90
total	1	15	4	14	9	4	0

Individual peptides were used in the indirect IgG assay. Shown are signal to noise (S/N) values for each peptide. A S/N value >10 is considered reactive.

For subsequent studies, the peptides 1, 2, and 4 were blended at equimolar amounts and tested in a single prototype HDV IgG assay. Specificity of the serology prototype assay was determined by testing uninfected donors from the US that were pre-screened for HBV (HBsAg, anti-HBc, and DNA), HCV (antibodies and NAT), HIV (antibodies and NAT) (n = 173). A provisional cut-off was determined based on the population median +20 standard deviations; and resulted in a specificity of 100%.

**Figure 4:**
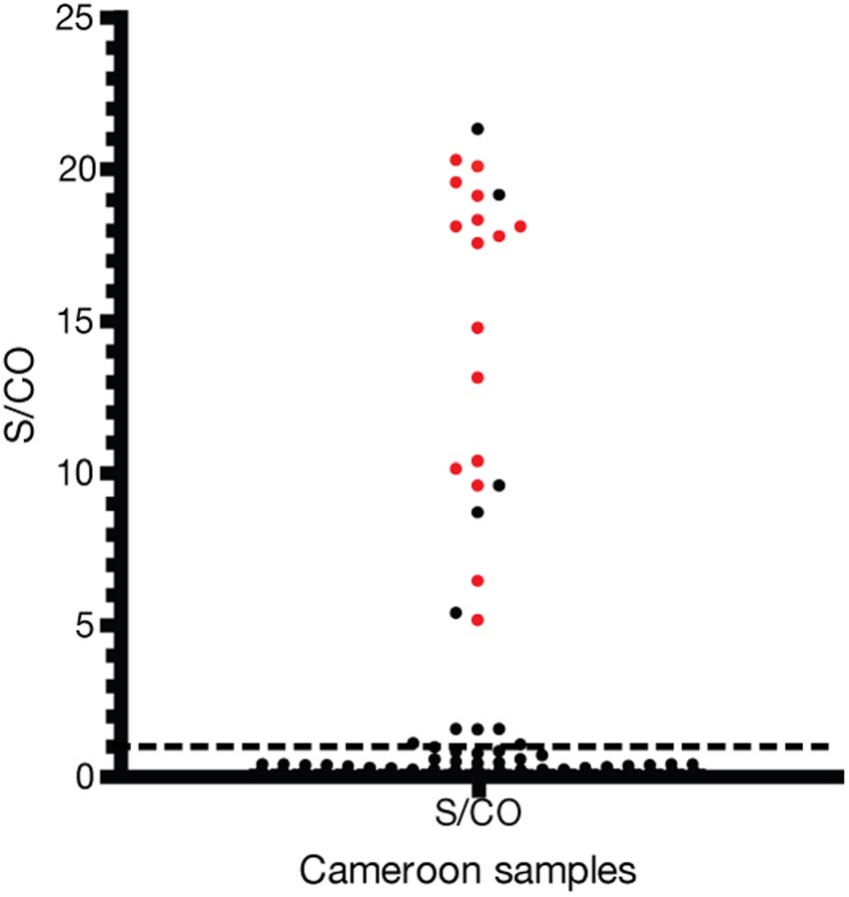
Scatterplot of anti-HDV IgG S/CO values for samples from Cameroon. S/CO values of Cameroon HBsAg (n = 145) samples tested with the prototype anti-HDV IgG assay. The cut-off (S/CO = 1.0) is indicated by the dotted line. Red circles indicated HDV RNA positive samples.

The complete 145 HBsAg positive sample set from Cameroon was tested with the prototype HDV IgG assay using the provisional cut-off. In total, 23 of 145 (16.0%) samples in this cohort were HDV IgG positive (Fig. 4), and all 16 HDV RNA positive samples had detectable antibodies resulting in a positive predictive value of 69.6% of the prototype test. Sero-reactivity of the sample set was confirmed with a comparator anti-delta IgG assay; the prototype assay and the commercial ELISA matched detection of all HDV RNA positive samples (Supplemental Table S3). The comparator assay detected 3 of 7 antibody only samples detected by the prototype assay. Of the remaining 4 non-confirmed samples, 3 were low S/CO reactive in the prototype assay (S/CO <1.50).

### Identification of linear epitopes in HDV large antigen

**Figure 5:**
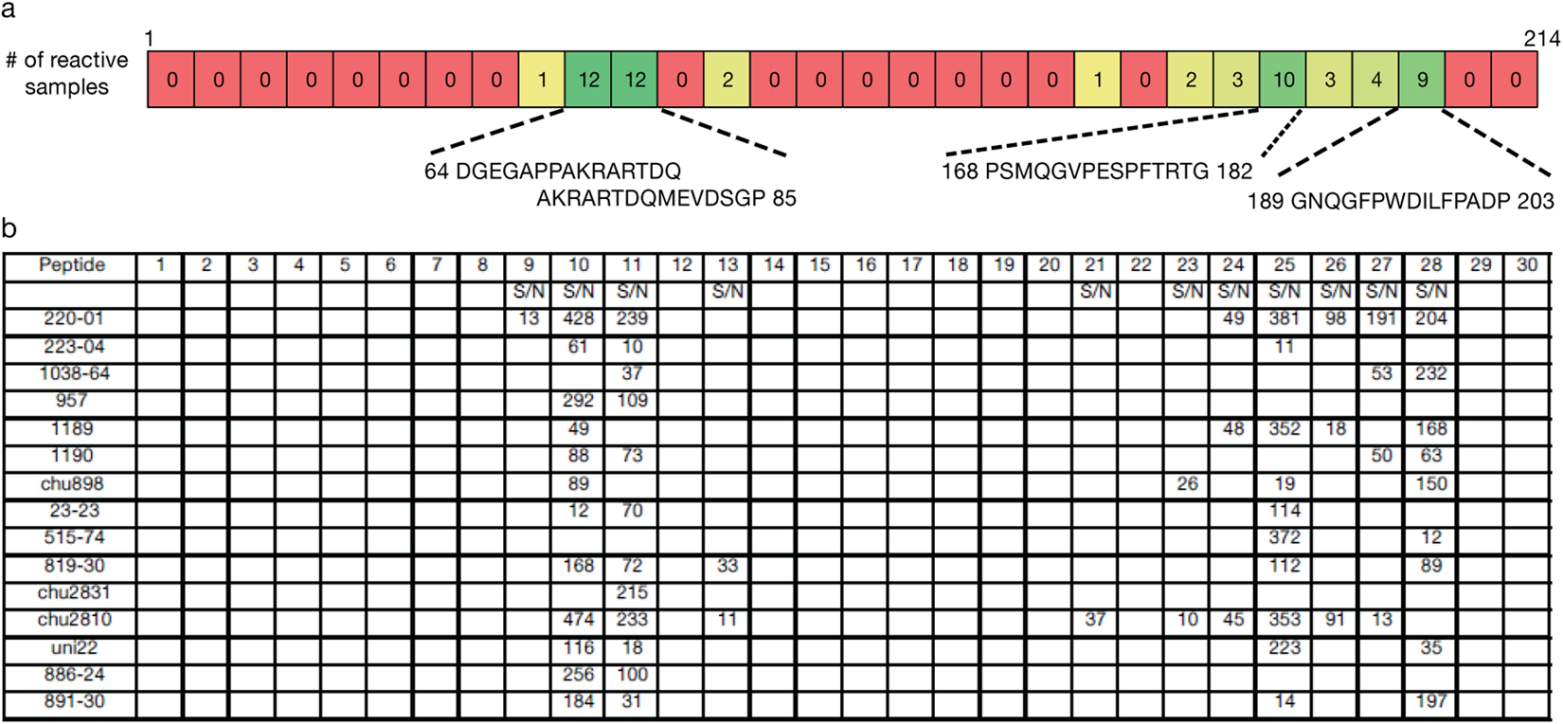
Linear epitopes identified using HDV large antigen scanning peptides. (**A**) Heat map analysis of reactivity of HDV RNA positive samples (n = 15) tested with individual scanning peptides in an indirect IgG assay. Indicated are the number of reactive samples for each scanning peptide and the amino acid residues of reactive peptides 10, 11, 25, and 28. Refer to Fig. 3 for complete amino acid sequence used in the peptide scan. (**B**) Shown only are S/N values ≥10; considered reactive.

Additionally, a peptide scan of the entire HDV large antigen open reading frame was designed to identify small linear epitopes. The study using the larger 7 peptides defined antigenic regions within amino acids 59–84 and 92–114. HDV RNA positive samples were tested with the scanning peptides in an indirect IgG assay (Fig. 5A). Two overlapping peptides (aa 64–85) showed reactivity with 14/15 samples tested (Fig. 5B); the sequence AKRARTDQ was common to both peptides and mapped to the original peptide 2 (Fig. 3). Additional linear epitopes were identified in regions (aa 168–182 and 189–203) that were not included in the original peptide design (Fig. 5A). The original peptide 4 detected several samples, but small peptides derived from the original peptide 4 were not reactive in the assay (Fig. 5A).

### Seroconversion sensitivity of prototype HDV molecular and serology assays

**Figure 6:**
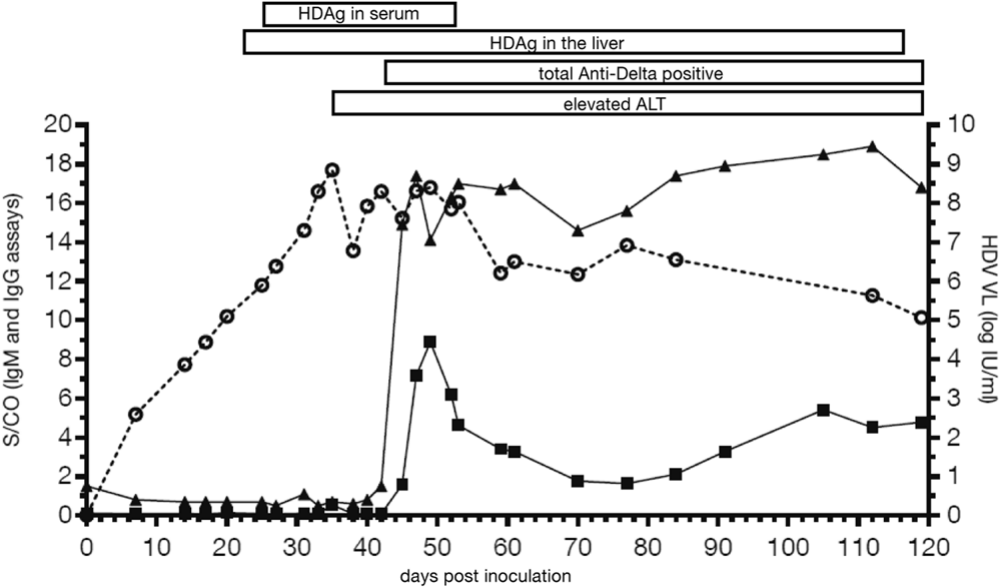
HDV superinfection progression to chronic HDV infection. Serial bleeds were tested for IgM and IgG specific responses using modified indirect assays. Plotted are S/CO values where cut-off was determined by taking 10 times the negative control for each assay (left Y-axis). S/CO ≥1.0 is considered reactive for both IgM and IgG assays. HDV viral load (log IU/ml) was determined using the Abbott HDV molecular assay (right Y- axis). Circle, viral load; square, IgM S/CO, triangle, IgG S/CO. Historic data is presented for HDAg in serum, HDAg in liver, elevated liver ALT, and total Anti-Delta (Abbott Anti-Delta EIA assay (3018)^25^. Presence of any of these markers is indicated by a solid box.

The molecular and serologic prototypes were used to test a serial bleed panel in order to determine the performance on samples that ranged from acute to established HDV infection. Previously characterized serial bleed samples were available from an HBsAg positive chimpanzee superinfected with HDV and collected for 119 days post infection^25^. Historic testing data showed detection of HDV antigen in the serum between days 25–52, whereas detection in in the liver occurred between days 27 – end of study day 119 (Fig. 6)^25^. Test results generated with the adapted HDV prototype for IgM and IgG indicated both IgM and IgG responses were detected at a similar time (days 45 and 42, respectively) compared to the historic testing data (day 42) using a FDA approved assay (Abbott Anti-Delta) which detected total antibody (IgM and IgG) (Fig. 6). The IgM response showed rapid decline after day 50, followed by a second wave of increased IgM levels between days 90 and 100 (Fig. 6). HDV RNA was detectable starting at day 7 and throughout the study (119 days total), and the IgG response began on day 45 and remained detectable throughout the study (Fig. 6). Viral titers ranged from 2.59 log IU/ml following infection to a peak of 8.85 log IU/ml around day 35, concomitant with ALT elevation. Viral titers declined upon the appearance of IgM and IgG antibodies, but were still detectable at 5.07 log IU/ml on day 119.

## Discussion

Prototype serologic and molecular tests for the diagnosis of HDV infection were developed for use on the high-throughput automated ARCHITECT and *m*2000 platforms, respectively. The HDV molecular prototype displays highly sensitive and reproducible detection of HDV RNA with a limit of detection of 5 IU/ml (Table 1). The prototype HDV serology assay is a sensitive and specific assay for the detection of anti-HDV IgG antibodies present during active and resolved HDV infections. Detection of antibody positive samples using the serology prototype had a positive predictive value of 69.6% for HDV viremia in a Cameroonian HBsAg negative sample set; however, the detection of HDV antibodies alone will not distinguish active from resolved infection. Infection with HDV is possible for all HBsAg individuals but routine testing worldwide for HDV is low. When used sequentially, the two prototype assays can provide high confidence in detection of HDV infection.

To confirm the HDV RNA positive results from the molecular prototype assay, the samples were tested with two established HDV RNA assays^24,20^. The comparator assays confirmed 12 of the 14 samples detected as positive by the prototype HDV RNA assay. The discordant samples had relatively low viral loads (<1.60 log IU/ml) in the prototype assay, and were anti-HDV IgG positive using the prototype serology assay (Table 5, Fig. 4), suggesting that these are true positives. All three HDV molecular assays targeted the conserved ribozyme region; but, assays differed in oligonucleotide sequences, probe complementarity, thermocycling conditions, and the addition of the detection enhancer reagent in the prototype assay to promote denaturation of RNA secondary structure (Supplemental Table S2).

Genetic diversity among HDV genotypes can impact detection by HDV molecular and serologic assays. Because of limited availability of samples representing the diversity of HDV, we challenged the prototype molecular assay using *in vitro* transcripts encompassing mismatches in the primers and probe regions identified in naturally occurring HDV isolates (Figs 1 and 2). Alignment analysis of sequences showed the regions targeted in the molecular prototype were highly conserved (269 of 291 sequences analyzed), although mutations were noted. The molecular assay detected all *in vitro* transcripts, but demonstrated a delay in detection of a transcript harboring a mismatch in both the forward primer and probe regions. The transcript was derived from Genbank accession number GU177144.1 which is a highly divergent unclassifiable genotype strain of HDV. From the alignment analysis, we note that the occurrence of several mismatches within the primers and probe targets was very rare, but our data show that this may result in a synergistic defect in the molecular prototype assay. Thus, while the molecular assay was able to tolerate mismatches, monitoring of sequence diversity as more sequence data is available will allow continued improvement of HDV detection.

The prototype serology assay was developed to detect the IgG response to HDV infection. The initial study used peptides spanning regions from the large antigen predicted to be antigenic, followed by a peptide scan to find discrete linear epitopes. The peptides 2 and 4 were designed to encompass non-overlapping regions (aa 59–84 and 92–114) and a combination of both detected all HDV RNA positive samples tested (Fig. 3 and Table 5). The peptide scan of the large antigen (Fig. 5) identified linear epitopes in 3 regions (aa 64–85, 168–182, and 189–203) and correlated well with areas that have been identified using HDV seropositive samples derived from woodchucks and humans^26–28^. The amino acid 64–85 region contains a nuclear localization signal and was previously to have utility in detecting HDV antibodies^26–28^. In addition, the length of the peptide was important for reactivity. Peptide 4 (aa 92–114) detected 14 of 16 RNA positive samples, but the smaller scanning peptides covering that region did not. The assay format using a blend of peptides is amendable to adding additional peptide variants as more diverse HDV strains are isolated.

Both prototype assays were used to test an archived HDV superinfected chimpanzee serial bleed panel in order to show detection from acute to established infection. The HDV viral load peaked around day 35 prior to seroconversion to IgM (day 45) and IgG (day 42); RNA was detectable from day 7 and remained detectable throughout the study (119 days total). The IgM and IgG responses developed at similar times to each other and to historic data, but the IgM response declined quickly while the IgG response remained, suggesting that testing for IgG only is sufficient as a primary screen. Consistent with this, all HDV RNA positive samples from the Cameroon cohort tested were IgG positive. Combining the seroconversion testing and the positive predictive value of an HDV IgG result for HDV RNA presence supports the importance of the anti-HDV IgG testing in detection of early phase HDV superinfection as well as established persistent HDV infection in HBsAg positive patients.

All patients with HBV are susceptible to HDV infection and testing should be a part of HBV monitoring^29^. Currently, as more HDV specific therapies emerge^30,31^ the need for highly specific and sensitive assays is evident. As the genetic diversity of HDV is better understood, additional updates to serologic and molecular assays will be needed. Diagnostic testing for HDV provides an opportunity for treatment and potentially improved clinical outcomes.

## Methods

### Alignment of HDV full length genomes

Full length genomes for HDV (>1680 nt) (Supplemental Table S1) were downloaded from Genbank and aligned using MUSCLE (MegAlign Pro, DNAStar, Madison, Wisconsin, USA). Alignments were manually edited in BioEdit version 7.0.4.1^32^ or higher to remove gaps. The primers and probe regions were identified in the alignment and point mutations were determined.

### HDV RNA assay

1.0 ml of plasma was extracted for total nucleic acids on the *m*2000*sp* using the open mode (RNADNA-BA-1000–55-v031113) protocol (Abbott Molecular, Des Plaines, IL). For RNA amplification, the oligonucleotides forward (400 nM, 5′-CTCCCTTWGCCATCCGAG-3′ W = A,T), reverse (400 nM, 5′-CTCTTCGGGTCGGCATGG-3′), and probe (200 nM, 5′-FAM-ATGCCCAGGTCGGACCRC-TAMRA-3′) were used with the AgPath ID One-Step RT-PCR kit (Applied Biosystems, Foster City, CA) mastermix including proprietary Detection Enhancer added according to the manufacturer’s protocol. 15 ul of the 50 ul eluate was then added for a total reaction volume of 50 ul. Amplification and detection were performed on the *m*2000*rt* (Abbott Molecular, Des Plaines, IL) under the following cycling conditions: 50 C, 45 min; 95 C, 10 min; 50X (95 C, 15 s; 65 C, 30 s; 60 C, 85 s). Sample viral loads were determined by plotting to a standard curve generated from quantified dilutions of the WHO HDV NAT standard (PEI code number 7657/12).

In order to validate positive results from the prototype RNA test, eluates were re-evaluated in qRT-PCR reactions performed according to the published protocols^20,24^ (Supplemental Table S2). The published assays, Assay 1^24^ and Assay 2^20^, provide listings of the oligonucleotides used in the respective HDV RNA assays.

### *In vitro* transcription

Sequences from 12 different HDV isolates encompassing the assay amplicon (to 341 nt) were appended with an upstream T7 promoter for *in vitro* transcription (Integrated DNA Technologies, Coralville, IA). Gene blocks were used as template in the MEGAscript T7 Transcription kit (Life Technologies, Carlsbad, CA) according to the manufacturer’s instructions. Transcripts were extracted with an equal volume of TRIzol (Life Technologies, Carlsbad, CA), and chloroform (Sigma, St. Louis, MO). The upper aqueous phase containing the transcripts was recovered and purified by twice passing through a NucAway spin column hydrated in water. Transcripts were quantified by determining A_260_ (DU800 Beckman Spectrophotometer) and evaluated using the prototype HDV molecular assay.

### Samples

The WHO HDV standard for nucleic acid amplification techniques (NAT)-based assays (PEI code number 7657/12) was acquired through the Paul-Ehrlich-Institut (Langen, Germany) and was reconstituted according to instructions per use in 0.5 ml of sterile nuclease-free water (Thermo Fisher Scientific, Waltham, MA).

Volunteer donor serum for specificity studies was sourced from the United States through purchase from ProMedDx, LLC (Norton, MA, USA) as negative for HBsAg, anti-HBc, HBV DNA, HCV (antibodies and NAT), HIV (antibodies and NAT). The specificity for the serology prototype was tested with 173 donor samples, where 68 had remaining volume available for specificity testing using the molecular assay.

HBsAg reactive plasma samples (n = 145) were selected from a well-characterized convenience population that was available retrospectively from blood donations collected in Douala and Yaoundé, Cameroon between 2006 and 2011^33^; the study protocol was approved by the National Ethics Committee of Cameroon and samples were collected in accordance with relevant guidelines and regulations. Informed consent was obtained prior to sample collection. HBsAg status of samples was determined using either the ARCHITECT HBsAg (1L80) or ARCHITECT HBsAg Qualitative (4P53) assays (Abbott Diagnostics, Abbott Park, IL, USA). HBV viral load was determined using the RealTime HBV viral load (2G34) (Abbott Molecular, Des Plaines, IL). This cohort was chosen for HDV assay development due to the availability of large sample volumes (>5 ml).

### Sensitivity, reproducibility, and linearity testing

The limit of detection (LOD) was established by testing the WHO HDV standard (PEI code number  7657/12) diluted in HBV/HCV/HIV negative human plasma to 1.25, 2.5, 5, 10, and 20 IU/ml and extracted as described above (Table 1). The lowest dilution at which >95% of replicates were detected was accepted as the provisional LOD (Table 1). Reproducibility and linearity of the assay were evaluated by testing 3 sets of 10-fold dilution series of the WHO HDV plasma standard (5–50,000 IU/ml) extracted as described above.

### Design of HDV peptides

A consensus HDV large antigen amino acid sequence was constructed using 230 sequences, predominantly genotype 1, from Genbank using MUSCLE (MegAlign Pro, DNAStar, Madison, Wisconsin, USA) (Supplemental Table S1). Amino acid conservation was determined using the MegAlign program (DNAStar, Madison, Wisconsin, USA). The consensus sequence was modeled using the Protean 3D program (DNAStar) to show areas of predicted secondary structure, hydrophobicity, and antigenicity for peptide design. A total of 7 peptides were designed to areas of predicted antigenicity across the entire 214 amino acids of the large antigen (Fig. 3). A peptide scan was made of the consensus sequence comprised of 15mers with 8 amino acid overlap. All peptides were ordered from Genscript (Piscataway, New Jersey, USA) as N-terminal biotinylated and C-terminal amidated.

### HDV serology assay

The HDV peptides were used in an indirect anti-human IgG assay format developed on the ARCHITECT immunoassay instrument (Abbott Diagnostics, Abbott Park, IL). Briefly, the biotinylated peptides were diluted at 400 ng/ml each and incubated with magnetic streptavidin microparticles and sample for 18 minutes (first step) to allow for immunocomplex formation. The magnetic microparticles were washed and an acridinium conjugated mouse anti-human IgG was added (second step) for detection of immunocomplexes specific for the HDV peptides. The 7 original peptides were tested individually with HDV RNA positive samples to evaluate serologic utility. After determining that three peptides (1, 2, and 4) had value in the detection of HDV RNA positive samples, subsequent testing for sensitivity and specificity was performed with the peptides blended at 400 ng/ml each in a prototype assay. Peptide 1 was included because it may provide value for peptide 2 non-reactive or weakly reactive samples (example 515–74, Table 5). A provisional cut-off for the prototype assay was determined after testing n = 173 volunteer donors (HBV negative) and determining the median relative light unit (rlu) count plus 20 standard deviations.

Scanning peptides were tested in an indirect IgG assay where 400 ng/ml of each peptide was mixed into pools of 5 in sequential order for testing. HDV RNA positive sample were tested and pools that showed reactivity (S/N >0) were dissected for individual peptide reactivity.

For serology assay comparison testing, the XpressBio HDV-IgG ELISA (Frederick, Maryland, USA) was used as a comparator assay for testing the HBsAg positive samples from Cameroon as per manufacturer’s instructions.

### Chimpanzee seroconversion model

An HBV infected chimpanzee (HBsAg positive) was experimentally superinfected with HDV (genotype 1) in 1984 using a standardized acute-phase inoculum provided by the National Institutes of Health (NIH), which had been derived from the plasma of a previously infected chimpanzee. All procedures were performed under institutionally approved protocols at the Laboratory for Experimental Medicine and Surgery in Primates (LEMSIP, New York University, Tuxedo, New York) in accordance with relevant guidelines and regulations in place at the time of the study, 1984^34–36^. Serial bleeds were collected for 119 days post superinfection and were originally used in a publication describing the assay performance of the Abbott Anti-Delta EIA (no longer commercially available) to detect acute HDV infection^25^. Briefly, both chimpanzee liver and serum HDAg were detected by using a modified research sandwich enzyme immunoassay^37^. Serum alanine aminotransferase (ALT) was measured by a commercial reference laboratory at the study site the day of the chimp bleed. Small volumes of the serial bleeds from the study were preserved at −80 °C in 1985 were used for testing herein. The prototype serology assay was modified where the conjugate used for detection was changed to either a goat anti-monkey IgG or goat anti-monkey IgM for use in the indirect assay format. Provisional cut-offs for IgM and IgG assays were set at 10 times the background (negative control) signal.

### Statistical analysis

Positive predictive value and P-value (unpaired student’s t-test) were determined using Prism (GraphPad Software Inc, La Jolla, CA). Regression analyses were performed in JMP (JMP 11.2.0, SAS Institute, Inc., 2013).

### Data Availability

Sequences deposited in Genbank under accession numbers (KY861350, KY861351, KY861352, KY861353, KY861354, KY861355).

## Electronic supplementary material

Supplemental Information
