# Author Correction: Development and performance of prototype serologic and molecular tests for hepatitis delta infection

**DOI:** 10.1038/s41598-020-62687-4

**Published:** 2020-03-31

**Authors:** Kelly E. Coller, Emily K. Butler, Ka-Cheung Luk, Mary A. Rodgers, Michael Cassidy, Jeffrey Gersch, Anne L. McNamara, Mary C. Kuhns, George J. Dawson, Lazare Kaptue, Birgit Bremer, Heiner Wedemeyer, Gavin A. Cloherty

**Affiliations:** 1 Abbott Laboratories, Abbott Park, IL USA; 2 Université des Montagnes, Bangangté, Cameroon; 3 Department of Gastroenterology, Hepatology and Endocrinology, Hannover Medical School, Hannover, Germany

Correction to: *Scientific Reports* 10.1038/s41598-018-20455-5, published online 01 February 2018

In this Article, the authors neglected to cite a previous paper. This reference is being added because it contains important information on how an RT-PCR analysis was performed. The reference is included below as Ref 1 and should be cited in the text as follows:

In Supplementary Table S4, in column 5 ‘Ref’, all instances of reference 35 should instead refer to this omitted reference.

In addition, in the Table legend of Supplementary Table S4:

“Primers and cycling conditions were previously described^35^”

should read:

“Primers and cycling conditions were previously described^1^”
