# Supplementary material for: Development and performance of prototype serologic and molecular tests for hepatitis delta infection
Source: Sci Rep. 2018 Feb 1;8:2095. doi: 10.1038/s41598-018-20455-5 (PMC5794785; doi:10.1038/s41598-018-20455-5)
Supplement: Supplementary file 1 — Supplemental Information [file 41598_2018_20455_MOESM1_ESM.pdf]

Development and performance of prototype serologic and molecular tests for hepatitis delta infection

Kelly E. Coller[a]*, Emily K. Butler[a], Ka-Cheung Luk[a], Mary A. Rodgers[a], Michael Cassidy[a], Jeffrey Gersch[a], Anne L. McNamara[a], Mary C. Kuhns[a], George J. Dawson[a], Lazare Kaptue[b], Birgit Bremer[c], Heiner Wedemeyer[c], Gavin A. Cloherty[a]

aAbbott Laboratories, Abbott Park, IL, USA.

b Université des Montagnes, Montagnes, Bangangté, Cameroon.

c Department of Gastroenterology, Hepatology and Endocrinology, Hannover Medical School, Hannover, Germany.

*Corresponding author: kelly.coller@abbott.com

Materials and Methods

Sequencing and phylogenetic analysis

Viral RNA was extracted from 0.5ml of plasma using the open mode (RNADNA-BA-1000-55-v031113) protocol (Abbott Molecular Diagnostics, Des Plaines, IL). Eluted RNA (10ul) was used as template for reverse transcription using random hexamers. The resulting cDNA was used as template for 3 nested PCRs using primers and cycling conditions described in [35]. PCR reactions were nuclease and protease treated prior to the sequencing PCR reaction using the primers (supplemental table 4). Sequencing reactions were prepared using the Big Dye Terminator Cycle Sequencing Ready Reaction kit v3.1 and electrophoresed on the ABI 3130xl Genetic Analyzer (Applied Biosystems, Foster City, CA). Near complete genome contigs were assembled and edited using Sequencher v5.4.1 (Gene Codes Corp., Ann Arbor, MI). Viral sequences were aligned with HDV sequences representing genotypes 1-8 from Genbank using MUSCLE (Sequencher v5.4.1, Gene Codes Corp., Ann Arbor, MI). Alignments were manually edited in BioEdit version 7.0.4.1 [32] or higher to remove gaps and Neighbor-Joining phylogenetic inference was performed using PHYLIP 3.5c (J. Felsenstein, University of Washington, Seattle, USA) as previously described [36]. Classifications were assigned for specimens with bootstrap support of 70 or greater; the relevant branches leading to classification had a bootstrap value of 100.

Supplemental figure legends:

Supplemental figure S1. Correlation of HDV molecular assays

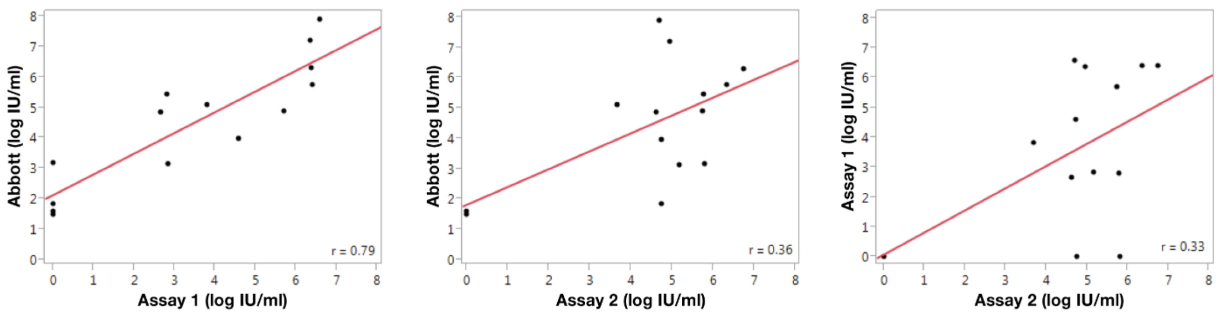

Comparison of HDV quantitation data across three molecular assays. Bi-variate analysis was performed using the calculated concentrations of each sample (log IU/ml) from the different assays. R2-values are given.

Supplemental table S1. Genbank accession numbers used in nucleotide and amino acid alignments

HDV full length genomes. Sequences with mutations in the primers and/or probe region are in bold.

AM779591.1 M58629.1 H LT604941.1 LT594477.1 KJ744237.1 KJ744234.1 KJ744217.1 KJ744215.1 KJ744216.1 KJ744243.1 KJ744244.1 KJ744247.1 KJ744248.1 KJ744254.1 KJ744257.1 KJ744226.1 KJ744225.1 KJ744255.1 KJ744249.1 AM779596.1 AM779585.1 KJ744218.1 **KJ744245.1** KJ744227.1 KJ744228.1 KJ744224.1 KJ744232.1 KJ744231.1 KJ744256.1 AM779593.1 AM779581.1 AM779582.1 AM902168.1 JX888098.1 JX888099.1 JX888110.1 JX888104.1 JX888112.1 M84917.1 H LT604950.1 LT604947.1 AM779575.1 KM110793.1 AM902181.1 AM779577.1 JX888111.1 JX888108.1 LT594479.1 AM902169.1 JX888105.1 AM902170.1 AM902167.1 LT703299.1 LT594476.1 AM779595.1 AM779584.1 AM779583.1 LT604951.1 LT604948.1 LT604949.1 KJ744214.1 KJ744253.1 AY261458.1 AY261460.1 AY261459.1 AY261457.1 AB118846.1 LT594481.1 KF660599.1 AF425645.1 AF104264.1 LT604952.1 X60193.1 JA417606.1 AX741209.1 AJ309879.1 LT604953.1 HW649777.1 JC493889.1 JA417607.1 AJ309880.1 AX741210.1 KF660598.1 **KM110804.1** **LT604972.1** LT594487.1 **KM110805.1** **KM110803.1** LT604969.1 JA417541.1 AJ584844.1 AX741144.1 **LT594486.1** **LT604971.1** **KM110802.1** **LT604970.1** **AM183333.1** LT604973.1 LT594488.1 **GU177114.1** LT604974.1 AM183330.1 JA417566.1 AJ584849.1 AX741169.1 **AM183327.1** LT604963.1 LT594483.1 LT604957.1 AM183331.1 LT604959.1 LT594482.1 LT594472.1 LT604961.1 LT604956.1 LT604960.1 LT604958.1 JX888106.1 JX888103.1 **JX888107.1** AM183328.1 JA417551.1 AJ584846.1 AX741154.1 JA417546.1 AJ584845.1 AX741149.1 LT604962.1 JA417556.1 AJ584848.1 AX741159.1 AM183326.1 LT604964.1 AM183329.1 JX888102.1 **AM183332.1** **LT594484.1** LT604968.1 JA417561.1 AJ584847.1 AX741164.1 LT594485.1 LT604967.1 AB118840.1 AB118833.1 AB118827.1 AB118818.1 **AF309420.1** AB118822.1 AB118831.1 AB118824.1 AB118823.1 AB118820.1 **AB118837.1** **AB118836.1** AB118829.1 AB118826.1 AB088679.1 AB118839.1 AB118838.1 AB118830.1 AB118828.1 AB118819.1 AB118835.1 AB118834.1 AB118832.1 AB118825.1 AB118821.1 AF018077.1 AY648952.1 AY648953.1 AY648954.1 AY648955.1 AB118843.1 AB118844.1 AB118842.1 AB118847.1 AF209859.1 AB118841.1 AB118845.1 AM779574.1 JX888101.1 JX888100.1 KM110796.1 AM902164.1 KM110790.1 AM779580.1 KM110791.1 LT594478.1 AM902180.1 AM902176.1 KM110801.1 KM110798.1 JX888109.1 KM110800.1 AM902171.1 LT594473.1 KM110795.1 JX888113.1 KM110794.1 KM110792.1 KM110797.1 KM110799.1 AM779592.1 U81989.1 AM902178.1 U81988.1 AM779576.1 KJ744242.1 KJ744240.1 KJ744241.1 EF514905.2 EF514907.1 EF514904.1 EF514903.1 LT604936.1 X85253.1 LT604944.1 LT604943.1 LT594480.1 AM779594.1 AM779586.1 AF098261.1 HM046802.1 AJ000558.1 JE954744.1 M21012.1 AJ307077.1 X04451.1 M55042.1 LT604938.1 **AM902175.1** KJ744250.1 KJ744235.1 KJ744233.1 LT604935.1 AM902177.1 AM902165.1 AM779587.1 AM779579.1 NC_001653. D01075.1 AM779578.1 AM902163.1 KJ744230.1 AM902179.1 AM779597.1 **M28267.1** AM902172.1 LT604937.1 KJ744238.1 KJ744229.1 L22066.1 AY633627.1 AM902174.1 AM902173.1 KY379246.1 KY379247.1 KF660602.1 KF660601.1 AB118849.1 AB118848.1 KF660600.1 X77627.1 KR363259.1 KR363258.1 KR363257.1 AY648958.1 HW649776.1 JC493886.1 AF104263.1 AF425644.1 AY648957.1 AY648956.1

M92448.1 H LT594475.1 LT604940.1 LT604939.1 AM902166.1 LT604942.1 AM779589.1 AM779588.1 KT722840.1 KJ744223.1 KJ744220.1 KJ744222.1 KJ744221.1 LT604945.1 KY495779.1 AM779590.1 LT604946.1

HDV large antigen sequences

AAQ09794.1 AGI51675.1 AAS18573.1 BAB68381.1 BAB68380.1 BAB68379.1 P0C6L3.1 AAP86460.1 ABO87232.1 ABO87231.1 ABO87230.1 ABO87229.1 ABO87228.1 ABO87227.1 ABO87226.1 ABO87225.1 ABO87224.1 ABO87223.1 ABO87222.1 ABO87221.1 ABO87220.1| ABO87219.1 ABO87218.1 ABO87217.1 ABO87216.1 ABO87215.1 ABO87214.1 ABO87213.1 ABO87212.1 ABO87211.1 ABO87210.1 ABO87209.1 ABO87208.1 ABO87207.1 ABO87206.1 ABO87205.1 ABO87204.1 ABO87203.1 ABO87202.1 ABO87201.1 ABO87200.1 ABO87199.1 ABO87198.1 ABO87197.1 ABO87196.1 ABO87195.1 ABO87194.1 ABO87193.1 ABO87192.1 ABO87191.1 ABO87190.1 ABO87189.1 ABO87188.1 ABO87187.1 ABO87186.1 ABO87185.1 ABO87184.1 ABO87183.1 ABO87182.1 ABO87181.1 ABO87180.1 ABO87179.1 ABO87178.1 ABO87177.1 ABO87176.1 ABO87175.1 ABO87174.1 ABO87173.1 ABO87172.1 ABO87171.1 ABO87170.1 ABO87169.1 ABO87168.1 ABO87167.1 ABO87166.1 ABO87165.1 ABO87164.1 ABO87163.1 ABO87162.1 ABO87161.1 ABO87160.1 ABO87159.1 ABO87158.1 ABO87157.1 ABO87156.1 ABO87155.1 ABO87154.1 ABO87153.1 ABO87152.1 ABO87151.1 ABO87150.1 ABO87149.1 ABO87148.1 ABO87147.1 ABO87146.1 ABO87145.1 ABO87144.1 ABO87143. ABO87142.1 ABO87141.1 ABO87140.1 ABO87139.1 ABO87138.1 ABO87137.1 ABO87136.1 ABO87135.1 ABO87134.1 ABO87133.1 ABO87132.1 ABO87131.1 ABO87130.1 ABO87129.1 ABO87128.1 ABO87127.1 ABO87126.1 ABO87125.1 ABO87124.1 ABO87123.1 ABO87122.1 ABO87121.1 ABO87120.1 ABO87119.1 ABO87118.1 ABO87117.1 ABO87116.1 ABO87115.1 ABO87114.1 ABO87113.1 ABO87112.1 ABO87111.1 ABO87110.1 AAG26089.1 AAG26086.1 AJO72442.1 AJO72441.1 AJO72439.1 AJO72438.1 AJO72437.1 AJO72435.1 AJO72434.1 AJO72433.1 AJO72432.1 AJO72429.1 AJO72428.1 AJO72427.1 AJO72426.1 AJO72425.1 AJO72423.1 AJO72422.1 AJO72421.1 AJO72420.1 AJO72419.1 AJO72417.1 AJO72416.1 AJO72415.1 AJO72412.1 AJO72410.1 AJO72409.1 AJO72408.1 AJO72407.1 AJO72406.1 AJO72405.1 AJO72404.1 AJO72403.1 AJO72402.1 AJO72401.1 AJO72400.1 AJO72399.1 AAT68020.1 AAU93913.1 ABP87998.1 ABP58677.1 ABP58676.1 ABP58675.1 ABP58674.1 AAC40216.1 CCU64152.1 CCU64151.1 CCU64150.1 ADI24879.1 CAJ66094.1 CAJ66092.1 CAE51164.1 CAE51159.1 P29996.2 P06934.3 P0C6M3.1 P0C6L6.1 P0C6M5.1 Q81835.2 P0C6L5.1 Q81842.2 P25989.2 P0C6M9.1 P29997.3 P0C6M7.1 P0C6M6.1 P0C6M8.1 P0C6M2.1 P0C6M1.1 P0C6L4.1 P29833.3 Q91DH9.3 Q91DH8.3 Q91DH7.3 P25880.2 P69618.2 P0C6M4.1 P0C6M0.1 P0C6L9.1 P0C6L8.1 P0C6L7.1 P25882.3 P25881.3 P25884.3 Q9E925.2 P69619.2 AAV65725.1 AAV65724.1 AAV65723.1 AAV65722.1 AAV65721.1 AAV65720.1 AAV65719.1 AAV65718.1 ADN94646.1 ADN94645.1 ADN94644.1 ADN94643.1 ADN94642.1

Supplemental table S2. Comparison of HDV assays

	Abbott	Assay 1 [24]	Assay 2 [20]
**LOD**	5 IU/ml	750 cp/ml	15 cp/ml
**LOD target**	Extracted WHO standard	Diluted RNA transcript	Diluted DNA plasmid
**PCR kit**	Ambion Ag-Path ID	Ambion Ag-Path ID	Qiagen QuantiTect Virus
**Detection enhancer**	included	excluded	N/A
**Thermocycling**	RT: 50C, 45min; 95C, 10min	RT: 45C, 10min; 94C, 10min	RT: 50C, 20min; 95C, 5min
	50X (95C, 15s; 65C, 30s; 60C, 85s)	45X (95C, 30s; 60C, 60s)	50X (95C, 15s; 60C, 65s)

Supplemental table S3. Comparator ELISA testing of HDV RNA positive samples

	ELISA S/CO	ARCHITECT peptide (1,2,4) S/CO
1134	not available	19.09
1038-64	14.48	13.10
220-01	17.68	20.06
223-04	14.09	5.13
819-30	18.16	10.36
886-24	17.58	18.07
891-30	17.64	9.56
CHU2810	16.86	20.26
CHU2831	17.25	18.29
UNI22	17.32	19.11
957	18.72	19.53
1189	18.35	14.73
1190	18.12	17.53
23-23	18.32	17.75
515-74	17.59	6.42
CHU898	17.71	10.11

Shown are S/CO values.

Supplemental Table S4. Primers used for HDV sequencing

Template	Product	Primer name	Sequence	Ref
cDNA	PCR1	320ds	CCAGAGRAMCCCTTCARCGAAC	35
		rv900	GTCCGACCTGGGCATCCG	35
cDNA	PCR2	710s	CGCCGGCTGGGCAACATT	35
		1302das	GGNTTCACCGACRAGGAGAG	35
cDNA	PCR3a	HDVfwd1262	CTTGTTCTCSAGGGCCTTCC	This work
		HDVrev462	CCCGGGATAAGCCTCACTC	This work
PCR1	Sequencing strand	320ds	CCAGAGRAMCCCTTCARCGAAC	35
		rv900	GTCCGACCTGGGCATCCG	35
		HDVfwd407	AAGHAAAGADAGCAACGGGGCTAG	This work
PCR2	Sequencing strand	710s	CGCCGGCTGGGCAACATT	35
		1302das	GGNTTCACCGACRAGGAGAG	35
		HDVrev1276	GCCCTCGAGAACAAGAAGAA	This work
		HDVfwd884	CATGCCGACCCGAAGAGGA	This work
PCR3a	Sequencing strand	HDVfwd1262	CTTGTTCTCSAGGGCCTTCC	This work
		HDVrev462	CCCGGGATAAGCCTCACTC	This work
		HDVfwd1433	GTTTCCCAGCCAGGGAT	This work
		HDVrev330	CTTCTGTTCGCTGAAGGG	This work

Primers and cycling conditions were previously described [35].
